# A robotic treatment delivery system to facilitate dynamic conformal synchrotron radiotherapy

**DOI:** 10.1002/mp.17750

**Published:** 2025-03-16

**Authors:** Micah J. Barnes, Nader Afshar, Taran Batty, Tom Fiala, Matthew Cameron, Daniel Hausermann, Nicholas Hardcastle, Michael Lerch

**Affiliations:** ^1^ Centre of Medical Radiation Physics University of Wollongong Wollongong Australia; ^2^ ANSTO Australian Synchrotron Clayton Australia; ^3^ Physical Sciences Peter MacCallum Cancer Centre Melbourne Australia; ^4^ Sir Peter MacCallum Department of Oncology University of Melbourne Melbourne Australia

**Keywords:** robotic, conformal, synchrotron

## Abstract

**Background:**

In clinical radiotherapy, the patient remains static during treatment and only the source is dynamically manipulated. In synchrotron radiotherapy, the beam is fixed, and is horizontally wide and vertically small, requiring the patient to be moved through the beam to ensure full target coverage, while shaping the field to conform to the target. No clinical system exists that performs both dynamic motion of the patient and dynamic shaping of the beam.

**Purpose:**

We developed and tested a new dynamic treatment delivery system capable of delivering conformal fields with a robotic patient positioning system for use on the Imaging and Medical Beamline (IMBL) at the Australian Nuclear Science and Technology Organisation, Australian Synchrotron.

**Methods:**

An industrial robotic manipulator was modified to enable dynamic radiotherapy treatments on IMBL. The robot, combined with a carbon‐fiber treatment couch‐top and a recently developed dynamic collimator, formed the basis of the new treatment delivery system. To synchronize the motions of the robot and collimator, a real‐time, hardware‐based event‐handling system was utilized. To test the system, a ball bearing in a medical physics phantom was treated with circular fields ranging from 5 to 40 mm in diameter and at treatment speeds from 2 to 50 mm $s^{-1}$. The position of the ball bearing was compared to the center of the circular fields and the positional and temporal accuracy of the treatment delivery system was assessed, and appropriate treatment margins for the system were determined.

**Results:**

The vertical position of the ball bearing varied with treatment delivery speed (−1.06to0.93mm) while the horizontal position remained consistent (−0.05to0.09mm). The time‐delay between the robot and the collimator remained consistent (−35.5msto18.5ms) at treatment speeds above $2\;{\mathrm{mms}}^{-1}$. Data at $2\;\mathrm{mm}s^{-1}$ was right at the edge of both the robot capabilities and the analysis technique, and had larger variations in timing (0.0msto57.9ms). Horizontal margins of 0.51mm and vertical margins of up to 2.3mm were calculated for the treatment delivery system.

**Conclusions:**

We have implemented the first robotic treatment delivery system for synchrotron radiotherapy treatments. The largest errors were observed in the direction of motion of the patient through the beam and with future improvements, can be reduced. The system was both accurate and repeatable and is ready to support future treatments on IMBL.

## 1 INTRODUCTION

Synchrotron facilities have long been supporting the development of novel treatment techniques, such as microbeam raditation therapy (MRT),^1^, ^2^ ultra‐high dose‐rate (FLASH) radiotherapy,^3^ and targeted nano‐particle therapies.^4^ MRT utilizes synchrotron light to produce fields comprised of quasi‐parallel micro‐width beamlets that maintain tumor control while sparing healthy tissues.^5^ Due to the ultra‐high dose rates associated with synchrotron sources (in the order of $1000\;\mathrm{Gy}s^{-1}$), further tissue sparing is also possible on account of the FLASH phenomena.^6^ Radio‐sensitization of tumors is also possible with the introduction of nano‐particles given the kilovoltage energy spectrum produced by synchrotron sources (up to 300keV).^4^ Notably, because synchrotron beams are vertically thin and horizontally wide, the patient must be vertically translated through the synchrotron beam in order to fully cover the target volume.^7^ Therefore, delivering radiotherapy treatments with small beams at ultra‐high dose rates with high precision in a nonclinical environment presents many technical challenges that require novel or unique solutions.^8^

Recently, a novel dynamic conformal collimator was developed on the Imaging and Medical Beamline (IMBL) at the Australian Nuclear Science and Technology Organisation Australian Synchrotron to explore complex field shapes and arrangements in synchrotron radiotherapy treatments.^9^ The collimator uses two semi‐circular slits to dynamically shape the synchrotron beam as the patient moves through the synchrotron beam, and supports a maximum treatment velocity of $50\;\mathrm{mm}s^{-1}$, where the treatment velocity is defined as the velocity at which the patient is moved through the synchrotron beam. To support the accurate positioning and dynamic motion of a patient through the synchrotron beam, the IMBL also has access to a 6 degree‐of‐freedom industrial robotic manipulator with a carbon‐fiber couch‐top. In this work, we aim to integrate this novel collimator with the robotic manipulator on IMBL for radiotherapy treatments.

Previously, treatment fields on IMBL were defined using tungsten or cerrobend blocks that were fixed to the patient positioning system.^10^ In this work, not only is the patient dynamically translated through the beam, but the beam is also being dynamically shaped. The combination of dynamic beam collimation with dynamic patient motion increases the complexity of the delivery system and requires synchronization of the two motions.

The use of robotic positioning systems for radiotherapy purposes is common in ion‐based therapies. Robotic positioning systems were originally used in ion‐therapy to circumvent the need for rotating gantry beamlines.^11^ The robotic positioning system allowed greater control of the patient position with respect to a fixed beamline; however, the advantage of ion beams is that the beam can be steered across the target while the patient position remains static during beam on.

Robotic arms are also used in x‐ray based systems, such as CyberKnife, where both the patient and the megavoltage x‐ray source are positioned with separate robotic arms.^12^, ^13^ The distinct advantage of this approach is that the source can be moved relative to the patient, as well as the patient relative to the source. During treatment, the source position is dynamically adjusted to compensate for patient motion while the patient positioning robot is kept static. Further, to minimize the lag between patient motion on the robot and compensation of the motion by the source, a predictive patient motion model is used.

However, in both ion‐therapy systems and CyberKnife, the patient positioning system is always kept static while the treatment beam is on, and in each case, the dynamic adjustments to the radiation source only serve to counter‐act patient motion. Unlike ion‐therapy and CyberKnife, Tomotherapy dynamically moves the patient through the treatment beam in order to fully cover the target, although it does not use a robot for the patient positioning system. In Tomotherapy, a thin radiation beam is modulated as it is rotated around the patient, and the patient is translated through the beam at the same time, along a single mechanical axis. Synchronization of the rotating gantry, beam collimation and couch movements is achieved, and quality assurance protocols have been developed to assess the synchronicity of system.^14^, ^15^

As such, no solution currently exists for a robotic positioning system where the patient is dynamically moved through the treatment beam in order to cover the target. Further, dynamic patient motion with a robotic treatment couch has not been demonstrated with dynamic beam collimation in treatments that utilize ultra‐high dose rates, where single fraction treatments are delivered in a matter of seconds.

In the current study, we investigate the first use of a robotic patient positioning system synchronized with dynamic beam collimation using synchrotron radiation for radiotherapy treatments. Using a geometric phantom, we aim to assess the accuracy and repeatability of treatment delivery using this system and determine additional margins that account for the geometric accuracy of the system.

## 2 METHODS

### 2.1 Treatment delivery system

#### 2.1.1 Robotic patient positioning system

Hutch 3B on IMBL is located 136m from the source, and is 10m long by 6.3m wide. The hutch houses a robotic patient positioning system comprised of an industrial robotic arm and a customized carbon‐fiber treatment couch‐top. The robotic manipulator is a KUKA (Augsburg, Germany) *KR 150 R2700 extra* connected to a KUKA *KR C4* control unit, and controlled by a Siemens PLC. The manipulator is rated for payloads up to 150kg with positional accuracies of ± 0.06mm. The payload must cover the weight of every item attached to the robotic manipulator, including the patient bed, the immobilization apparatus, and the patient themselves. The couch‐top is designed for the imaging and treatment of large animals and is designed to mount to the robotic arm; it is complete with indexing markers and a grid of holes for attaching immobilization equipment.

**FIGURE 1:**
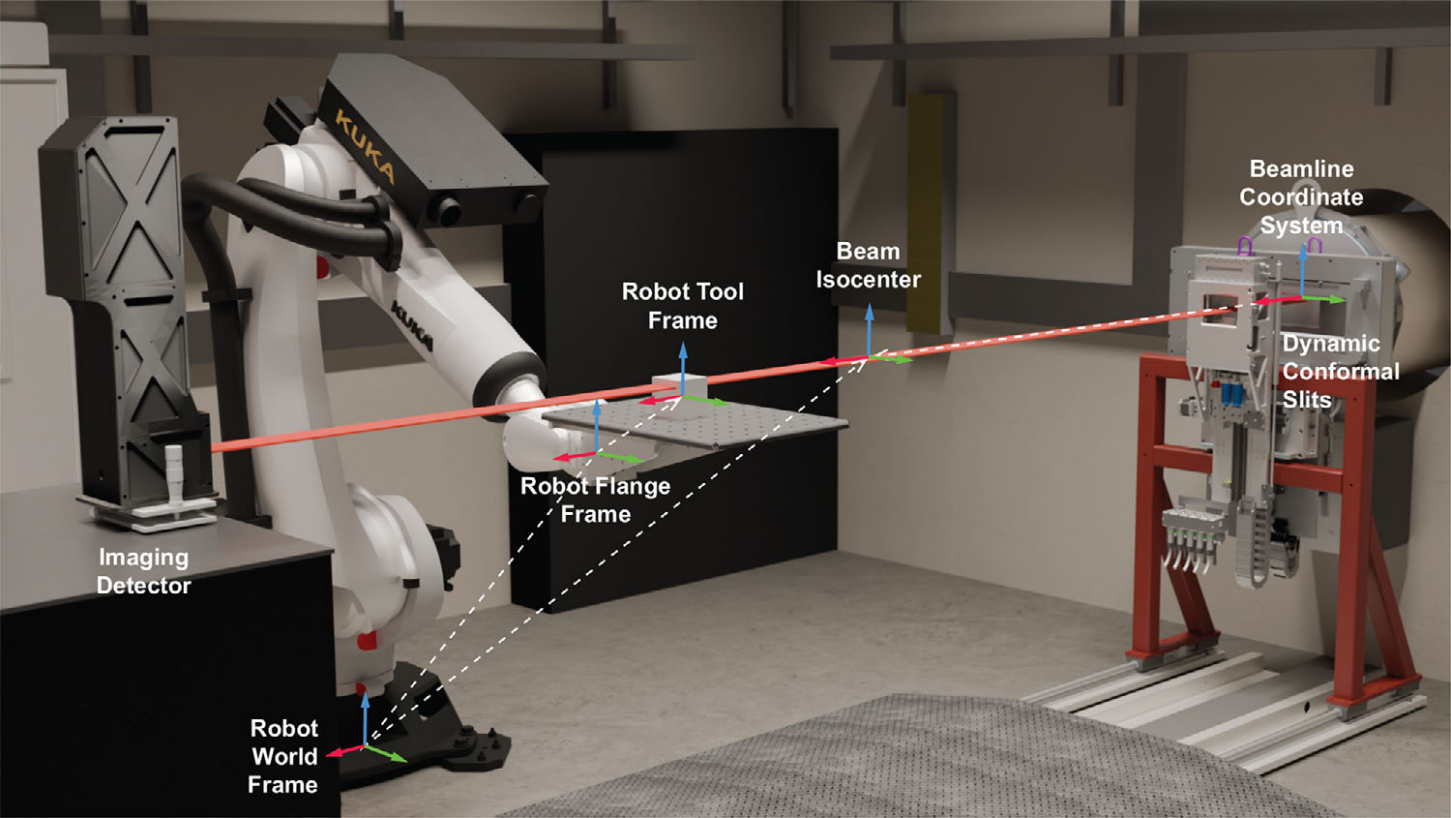
A rendering of the components and coordinate systems that make up the treatment delivery system in Hutch 3B on IMBL is shown. For reference, the distance between the beamline and beam isocenter coordinate systems is 3m. The same beam source is used for both imaging and treatment. The dynamic collimator is lowered out of the beam for image guidance, and is raised back into the beam for treatment. The coordinate systems are mapped from the beamline coordinate system (the nominated origin) through to the robot tool frame (patient position on the robot). IMBL, imaging and medical beamline.

The alignment of the robot to the beamline coordinate system is handled using frames and is illustrated in Figure 1. The beamline coordinate system is configured so that the origin is at the beam entrance into the hutch (x‐axis), and in the horizontal (y‐axis) and vertical (z‐axis) center of the beam. The beam isocenter is located 3m downstream from the beam origin. The robot world frame is located at the base of the robot and was calibrated at the time of installation; it is aligned with the beamline coordinate system orientation to within 0.005 degrees. The robot flange frame represents the end of the physical robot arm, relative to the world. The robot tool frame is located at a nominal position on the treatment couch, and is relative to the flange. The robot tool frame is updated to reflect the target position during patient alignment and treatment. The robot position is then calculated as the position of the robot tool frame relative to the beam is or, more importantly, the target position relative to the beam isocenter.

When considering a patient on the robot, the patient is first positioned on the treatment couch and is roughly aligned to the synchrotron beam using in‐room lasers. Then, the robot tool frame is configured to reflect this position, and the robot tool frame/patient position may be later refined using x‐ray based image guidance techniques.

When moving the patient, a constant velocity and orientation is defined for the duration of the movement. The kinematic properties of the motion (position, velocity and acceleration) are defined at the robot tool frame, such that they apply directly to the target. The KUKA *KR C4* control unit currently allows a constant velocity target between $2\;\mathrm{mm}s^{-1}\;\mathrm{to}\;2000\;\mathrm{mm}s^{-1}$. In Tomotherapy, patient velocity and acceleration are limited to $50\;\mathrm{mm}s^{-1}$ and $98\;\mathrm{mm}/\;s^{2}$ respectively.^16^ Alternatively Allgower et al.,^17^ state that the velocity and acceleration at the Midwest Proton Radiotherapy Institute are limited to $400\;\mathrm{mm}s^{-1}$ and 0.5g respectively, and they had not had any safety incidents with the robot at the time of publication. Therefore, in our setup, we have limited velocity targets to $50\;\mathrm{mm}s^{-1}$ and acceleration to $100\;\mathrm{mm}/\;s^{2}$, ensuring adequate patient safety and comfort.

Further, Wu et al.^18^ measured the path accuracy of a similar model KUKA robot when following a circular trajectory. They found that the standard deviation of the robot position from the path was 0.2mm in the plane of motion, and was 0.03mm tangent to the plane of motion (in line with the 0.06mm static positional accuracy). In this work, this would correspond to a standard deviation of 0.2mm in the vertical axis, and 0.03mm in the horizontal axis. They further observed that the largest deviations occurred during acceleration and deceleration regions, and highlighted that smooth acceleration profiles reduce jerk in the system, resulting in better positional control.

#### 2.1.2 Synchrotron beam

IMBL is a fixed beamline that produces an x‐ray beam of energies up to 300 kVp. In Hutch 3B, the usable beam size for radiotherapy treatments is approximately 3mm tall by 50mm wide with minimal roll‐off.^19^ In this work, the intrinisic synchrotron beam size was defined as 1mm tall by 55mm wide, the extra width was included to avoid clearance issues with the dynamic conformal collimator and the edge of the field.^9^ For imaging purposes, a wiggler field of 3T with a standard filtration of 4mm of aluminium and a monochromatic energy of 50keV was chosen. Further, because Hutch 3B is located 136m from the synchrotron source, the effects of beam divergence for both treatment and imaging are negligible.

#### 2.1.3 Imaging detector

The RUBY imaging detector (pictured in Figure 1) was used for its large dynamic range, high spatial resolution and fast frame rate capabilities.^20^ The imaging detector consists of a scintillator screen, an adjustable optical macro‐lens, and a pco.edge sCMOS camera (Excelitas Technologies Corp., Pittsburgh USA). The camera has a 2560 by 2160 pixel sensor (width by height), providing a maximum frame rate of 30Hz at full resolution. The detector was configured with a pixel size of 23.1μm by 23.4μm, resulting in a full resolution of 59.1mm by 50.5mm. However, since the synchrotron beam is vertically small, the camera's readout area was reduced to 59.1mm by 1.5mm (2560 by 64 pixels), fully capturing the synchrotron beam and allowing the frame rate to be increased to 100Hz. The detector was centered on the synchrotron beam and was kept in‐place for the duration of the experiment.

#### 2.1.4 Dynamic conformal collimator

The dynamic conformal collimator was recently developed on IMBL to enable shaping of the intrinsic synchrotron beam during radiotherapy treatments, and was designed to support treatment velocities of up to $50\;\mathrm{mm}s^{-1}$.^9^ The collimator was configured following the method in Barnes et al.^9^ Briefly, the collimator was first coarsely aligned to the synchrotron beam, and then each slit was carefully homed and centered on the synchrotron beam to within ± 23.4μm (one pixel size of the imaging detector). Collimator fields were modeled and pre‐calculated using the custom developed motion control software described in Barnes et al.^9^

### 2.2 Image guidance and patient alignment

Unlike Hutch 2B, Hutch 3B does not have an x‐ray tube for imaging purposes. Instead, a dynamic imaging technique was used to acquire large‐field images.^9^ Briefly, the patient was vertically translated through the beam while the detector captured images at 100 frames per second. Importantly, neither the detector or the beam moved during image acquisition, only the robot was vertically translated through the beam. The collected frames were then vertically stitched into a single, large‐field, planar x‐ray image. For more information on the dynamic imaging procedure, readers are invited to read Barnes et al.^9^

The x‐ray image was then imported into SyncMRT, an image guidance software developed for IMBL.^10^ Additional information about SyncMRT and the image guidance protocols can be found in Barnes et al.^10^ Using SyncMRT, the position of the target was identified relative to the synchrotron beam, and a transformation matrix that brings the target into alignment with the beam was calculated. The transform could not be applied directly to the robotic manipulator, thus it was split up into its translational and rotational components. The translational component was applied directly to the robot tool frame, centering the target on the beam. The position of the tool frame was also adjusted to reflect the new position of the target. The rotational component of the transform was then applied, as the center of rotation was closely aligned with the center of the target.

This image guidance routine was repeated until the target was within the alignment tolerance for the treatment. Since only a single field was utilized for treatment delivery, 2D image guidance was sufficient for alinging the target to the Beam's Eye View.

### 2.3 Treatment delivery synchronization

In previous treatments on IMBL, a pre‐fabricated tungsten block has been connected directly to the drive motor when translating the patient through the beam.^7^ By having the block attached to the patient, the beam could be turned on such that it dwells on the block first, the patient and block then translated through the beam with a single motor, and the beam turned off, without any dose extending beyond the shape of the block. Here, the dynamic collimator is completely separated from the robot, and thus both systems must be synchronized to carry out the treatment delivery. In order to synchronize the systems, we have used Zebra (Quantum Detectors, Harwell Oxford UK), a real‐time, hardware‐based event‐handling system with a clock time of 50MHz.^21^

**FIGURE 2:**
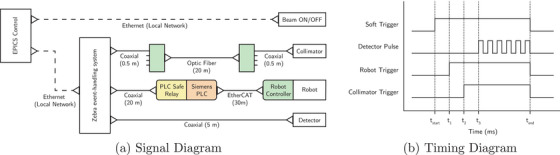
(a) The signal diagram depicts both the software signaling for beam ON/OFF and the connection of each component to the Zebra event‐handling system. (b) The timing diagram depicts the timing and structure of the signals sent from the Zebra event‐handling system to each connected component shown in (a). Note that the signals in (b) are not to scale and exist to demonstrate the signal structures only.

The Zebra system was configured to send trigger signals to the dynamic collimator, the robot controller, and the imaging detector. The Zebra triggers were configured to use TTL signals with a variable delay on each signal. The triggering configuration for each device is shown in Figure 2. As stated previously, collimator fields were modeled and pre‐calculated before treatment delivery. In addition to the collimator trajectories, the robotic patient positioning system was also pre‐calculated, such that the two systems could be moved to the appropriate starting points for treatment delivery and await a trigger signal to start their respective motions.

For imaging, a pulsed signal with a 5ms rising pulse and a 10ms period was sent to the detector to trigger frame capture at 100Hz. Each pulse resulted in the capture of one frame, where each frame had an acquisition period of 9.9ms, with 0.1ms left for frame read‐out.

**FIGURE 3:**
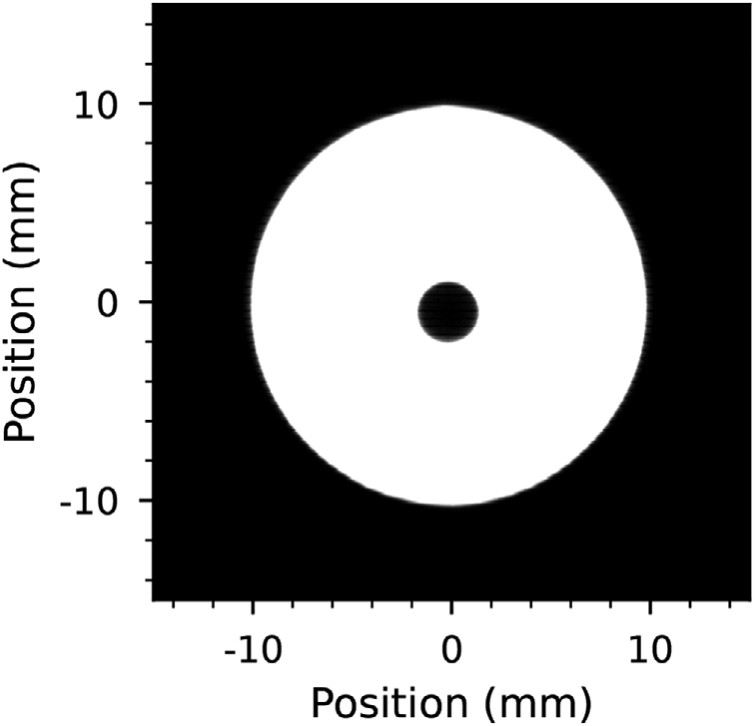
The alignment of a ball bearing relative to the center of a treatment field is shown using the dynamic image capture method described in Section 2.2. The image was formed by vertically stitching the frames captured by the imaging detector.

Finally, to calibrate the trigger delays for each system the delay was initialised at 0ms and a sample image of a ball bearing in a 20mm diameter field was acquired at $20\;\mathrm{mm}s^{-1}$. An example image is shown in Figure 3. The offset between the center of the field and the center of the ball bearing was measured as + 3.460mm, 173ms ahead of the treatment field. To compensate, the 173ms delay was applied to the collimator trigger signal, therefore aligning the motions of the collimator and robotic patient positioning system.

### 2.4 Experimental design

#### 2.4.1 Treatment planning

A series of treatment plans were generated for combinations of different sized circular fields and treatment delivery speeds. The circular fields were planned with diameters of 5, 10, 20, 30, and 40mm. The treatment delivery speeds were planned for 2, 10, 20, 30, 40, and $50\;\mathrm{mm}s^{-1}$. Fields were planned and simulated using the modeling software described in Barnes et al.^9^ The software models both patient movement and the operation of the dynamic collimator, simulates the treatment and provides the expected output field. In our previous study, we extensively validated the software model of the dynamic collimator. In this work, we extended the capabilities of the system by feeding the kinematic properties of the robotic positioning system into the algorithm.

For treatment delivery, the dynamic collimator was set to follow the trajectory of a target on the robotic positioning system. The robot trajectory was planned to start from rest (above the synchrotron beam) and accelerate at $100\;\mathrm{mm}/\;s^{2}$ until the desired velocity was reached, then finish at rest (below the synchrotron beam) by decelerating at the same rate. The ideal collimator positions were then calculated for each position in the robot trajectory. The individual motor trajectories in the collimator system were optimized separately (using the methods in Barnes et al.^9^) until a deliverable trajectory was obtained. The time‐synchronized motions of the robot and the collimator were simulated and the resulting fields were rasterized to a 0.25mm square grid.

#### 2.4.2 Target positioning

Treatments were planned on a customized image guidance phantom, a 100 × 100 × $100\;\;{\mathrm{mm}}^{3}$ acrylic cube with four internal 3mm diameter ball bearings,^10^ similar to the ISO Cube (CIRS Inc, Norfolk, VA, USA) phantom. The phantom was placed on the robotic positioning system, and aligned to the in‐room lasers using the external alignment markers on the phantom. Using x‐ray based image guidance, the chosen ball bearing for treatment was aligned to the center of the synchrotron beam. Once aligned, the phantom remained in place on the robotic positioning system for all treatments, and each of the planned treatments were carried out successively without removing the phantom.

#### 2.4.3 Field delivery

For each planned treatment, an automated delivery sequence was performed. The delivery sequence was as follows:

1. The pre‐planned trajectories for both the robotic positioning system and dynamic collimator were loaded onto their respective motion controllers.
2. The imaging detector was configured to capture the required number of images at the target frame rate of 100Hz.
3. The Zebra event‐handling system was configured with the appropriate signals to synchronize the treatment delivery and imaging timings.
4. All the motors in the treatment delivery system were initialized and checked for errors.
5. All motors were moved to their starting positions. The collimator started in a closed state, the patient was positioned above the synchrotron beam.
6. The beam was turned on and the Zebra event‐handling system began the timed triggering sequence for treatment delivery: all trajectories (patient motion and beam collimation) were delivered and the image frames were captured.
7. Once all motors reached their final resting positions, the beam was turned off. The collimator finished in a closed state, the patient was positioned below the synchrotron beam.

This was repeated for every planned field; the phantom remained in place on the robotic positioning system for the duration of all treatments.

### 2.5 Field analysis

Each delivered field, as captured by the imaging detector, was compared to its respective planned field. The center of the planned field was known, as each field was centered on the beam origin. The center of the delivered field, as captured by the imaging detector, was measured by taking the full‐width at half‐maximum (FWHM) of the top, bottom, left, and right sides of the circular field. The FWHM was again used to measure the center of the ball bearing within the delivered field. The difference between the center of the ball bearing and the center of the field was calculated.

A secondary analysis was carried out on the delivered fields, comparing the observed ball bearing size (width and height) to the physical size, and the delivered field size (width and height) to the planned size.

### 2.6 Treatment margins

The margin recipe by van Herk et al.^22^ was used to formulate treatment margins for the presented treatment system. Importantly, we only consider the geometric uncertainties resulting from patient positioning and treatment delivery, as this is the subject of this work. The presented margins do not consider additional sources of uncertainty such as patient motion, tumor contouring, or biological considerations.

The van Herk recipe takes the form

(1) $$m=2.5\Sigma +1.64(\sigma -\sigma_{p})$$

where Σ is the standard deviations of systematic errors, σ is the standard deviation of random errors, and $\sigma_{p}$ is the penumbra between the 95% isodose lines of the blurred and planned dose distributions.

Importantly, in synchrotron radiotherapy, treatments are often between one to three fractions,^23^ and can have up to five fields.^24^ Since the initial recipe does not accurately calculate margins for treatments with a small number of fractions, a residual error term is added to both the systematic and random errors.^25^ This results in two effective uncertainty terms ($\Sigma_{\text{eff}}$ and $\sigma_{\text{eff}}$) that compensate for the number of fractions (n) delivered:

(2) $$\Sigma_{\text{eff}}=\Sigma +\sigma /\sqrt{n}$$

(3) $$\sigma_{\text{eff}}=\sigma \left(1-1/n\right)$$

These have been validated for treatments containing one or more fractions.^26^ In this work, we consider the most common scenario in synchrotron radiotherapy, where only one fraction is delivered (n=1), reducing the residual random error to zero, and the random errors are instead fully included in the systematic error term.

Further, the recipe is often cited for MV linac‐based treatments where the beam penumbra is in the order of millimeters or centimeters depending on the treatment modality. In synchrotron radiotherapy, the kV energies result in an 80% ‐20% beam penumbra in the order of 0.3mm, and distance to the 95% isodose line is in the order of 0.1mm. For a single fraction, as is common in synchrotron radiotherapy experiments, we consider $\sigma_{p}$ to be 0.1mm. The values 2.5 and 1.64 are chosen to produce 3D margins that ensure a minimum CTV dose of D95% to 90% of the population.

## 3 RESULTS

### 3.1 Field analysis

A total of 531 fields were delivered and analyzed. The ball bearing size in all delivered fields was measured and found to agree with the physical size of the ball bearing in the phantom to within one pixel size (0.25mm). Further, the delivered fields also agreed with the planned fields to within one pixel size (0.25mm). This indicates that the robotic manipulator was reliably and consistently traveling at the requested delivery speed, and the dynamic collimator was accurately delivering the requested fields.

**FIGURE 4:**
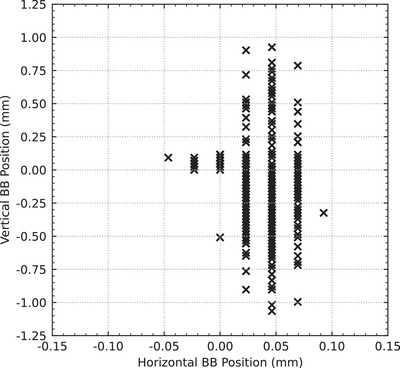
The scatter plot shows the difference between the center of the field and the center of the ball bearing in both the horizontal (y) and vertical (z) axes.

The position of the ball bearing relative to the center of the field for all fields is shown in Figure 4. In all delivered fields, the horizontal position of the ball bearing relative to the center of the treatment fields remained stable at −0.05to0.09mm (0.03 ± 0.02). Larger variations were observed in the vertical position ranging from −1.07to0.93mm (‐0.12 ± 0.31).

**FIGURE 5:**
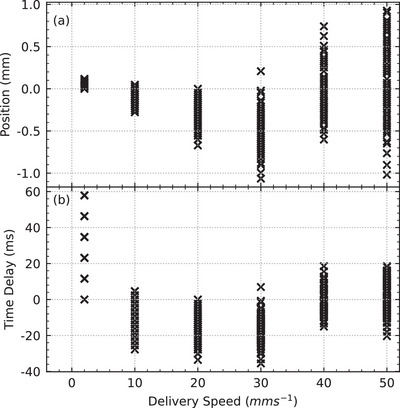
The vertical alignment of the ball bearing to the field from Figure 4, grouped by treatment delivery speed, is shown. (a) The veritcal offset is shown in millimeters. (b) The veritcal offset data in (a) is divided by the treatment speed to obtain the time‐delay between the positioning system and the dynamic collimator.

Figure 5a shows the vertical position of the ball bearing relative to the field center grouped by delivery speed, and Table 1 shows that the mean positional accuracy decreases for treatment speeds up to $30\;\mathrm{mm}s^{-1}$ and then improves for $40\;\mathrm{mm}s^{-1}$ and $50\;\mathrm{mm}s^{-1}$. This distance corresponds directly to the positional difference between the robotic positioning system and the dynamic collimator. When the difference is positive, the robot is lagging behind the collimator; when it is negative, the robot is ahead of the collimator. As such, by dividing the distance by the constant treatment velocity, the time‐delay between the two systems is obtained, this is shown in Figure 5b. Table 1 shows that the mean time‐delay varied from −15.6 to 30.0ms, but the standard deviation remained consistent at approximately 8ms across all treatment speeds, with the exception of the $2\;\mathrm{mm}s^{-1}$ data. Importantly, the difference in mean values between $10\;\mathrm{mm}s^{-1}\;\mathrm{to}\;50\;\mathrm{mm}s^{-1}$ treatment speeds is within one to two imaging frames (at 100Hz or 10ms).

**TABLE 1:** The min, max, mean, standard deviation and number of samples are presented for the vertical alignment of the ball bearing to the field as presented in Figure 5.

Speed ($\;\mathrm{mm}s^{-1}$)	Units	Min	Max	Mean	Std	*n*
2	mm	0.00	0.12	0.06	0.03	100
10	mm	−0.28	0.05	−0.11	0.07	100
20	mm	−0.67	0.00	−0.31	0.15	90
30	mm	−1.06	0.21	−0.46	0.25	80
40	mm	−0.60	0.74	−0.01	0.32	63
50	mm	−1.02	0.93	0.05	0.42	98
2	ms	0.0	57.9	30.0	13.9	100
10	ms	−27.8	4.6	−10.5	7.4	100
20	ms	−33.6	0.0	−15.7	7.3	90
30	ms	−35.5	6.9	−15.5	8.2	80
40	ms	−15.0	18.5	−0.1	8.0	63
50	ms	−20.4	18.5	0.9	8.4	98

While the positional difference for the $2\;\mathrm{mm}s^{-1}$ in Figure 5b were the smallest (< 0.12mm), the corresponding values when converted to a time delay in ms were the largest (up to 60ms). The time delay data is grouped into approximately 12.5ms bins, a direct result of the 0.25mm pixel size used in the field analysis. The time delay for the $2\;\mathrm{mm}s^{-1}$ group should therefore be analyzed with care as it is at the limit of the analysis technique.

### 3.2 Treatment margins

The uncertainty sources for our system are presented in Table 2, and are added in quadrature to form Σ and σ. Since there are obvious differences in the horizontal and vertical accuracy of our system, both Σ and σ have been defined as 3D vectors, such that the vertical and horizontal components are separately considered. The uncertainty sources presented in Table 2 only consider the geometrical components relating to treatment delivery, and excludes additional sources relating to a real patient such as imaging, tumor delineation and immobilization or positioning.

**TABLE 2:** The sources of uncertainty for both systematic (Σ) and random (σ) errors are presented in three dimensions. Each set of errors are added in quadrature and utilized in Equation 1. The treatment velocity (v) is in $\;\mathrm{mm}s^{-1}$. The x and y axes are the horizontal axes and the z axis is the vertical axis.

Source	Uncertainty type	Standard Deviation (mm) (*x*,*y*,*z*)	Reference
Treatment synchronization	Σ	(0.00, 0.00, 0.018v)	Table 1
Robot accuracy (static)	Σ	(0.06, 0.06, 0.06)	Manufacturer specifications
Robot accuracy (dynamic)	σ	(0.03, 0.03, 0.20)	Wu et al.^18^ Table 4
Conformal mask accuracy (dynamic)	σ	(0.26, 0.26, 0.26)	Barnes et al.^9^ Table 1

**FIGURE 6:**
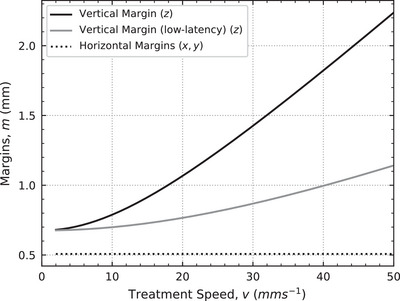
The horizontal (x,y) and vertical (z) treatment margins are plotted for treatment speeds from $2\;\mathrm{mm}s^{-1}\;\mathrm{to}\;50\;\mathrm{mm}s^{-1}$. The horizontal margins remain constant while the vertical margins are calculated as a function of treatment speed.

Because the synchronization between the robot and collimator systems is governed by the time‐delay between them, the treatment synchronization uncertainty presented in Table 2 is therefore presented as a function of the observed time‐delay (standard deviation of 18.8ms) multiplied by the treatment velocity. The effect of treatment velocity on margins is demonstrated in Figure 6, where sub‐millimeter margins at $2\;\mathrm{mm}s^{-1}$ increase to 2.3mm margins at $50\;\mathrm{mm}s^{-1}$. However, if the standard deviation of the time‐delay could be reduced to 8ms, as demonstrated for delivery speeds > $2\;\mathrm{mm}s^{-1}$ in Table 1, then the vertical margins could be reduced by up to 50 %. For example, at the fastest delivery speed of $50\;\mathrm{mm}s^{-1}$, the margin could be reduced from 2.3mm to 1.1mm (see low‐latency vertical margin in Figure 6).

The accuracy of the robotic manipulator during dynamic motion is described for our robot and control system in Wu et al.^18^, where reduced accuracy is reported for motion along a path (or in a plane) compared to motion perpendicular to the plane. Further, the conformal mask accuracy is defined as the standard deviation of the previously reported Hausdorff Distance, the maximum deviation of the field produced by the collimator from the planned field.^9^

**FIGURE 7:**
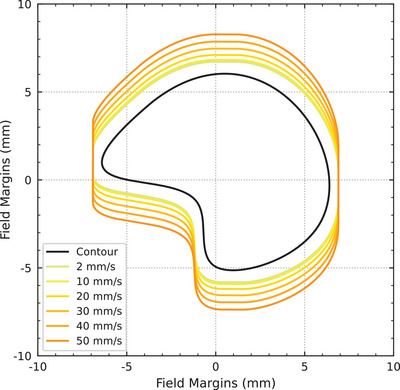
An example contour with calculated margins per treatment speed is presented. Margins were calculated using Equations 1–3.

The treatment margins as a function of treatment velocity are illustrated in Figures 6 and 7. Finally, margins are not provided at treatment velocities below $2\;\mathrm{mm}s^{-1}$ as this below the minimum velocity of the robot positioning system.

## 4 DISCUSSION

In this work, we integrated an industrial robotic manipulator and a novel dynamic collimator into a new treatment delivery system on IMBL. We used a hardware‐based event‐handler to synchronize the motion of each system for treatment delivery. We demonstrated that the synchronization between the two systems has clearly defined systematic and stochastic errors. The systematic error was demonstrated in Figure 5 and Table 1 by the floating mean value of the time‐delay between fields delivered at different treatment velocities. The stochastic error was demonstrated by the consistent standard deviation between the same groups. For basic, fixed‐velocity, linear treatment deliveries, the current setup offers modest treatment margins in the order of 0.5to2.3mm.

The existing small animal treatment capabilities on IMBL currently offer sub‐millimeter alignment under image guidance, with conformal fields (using pre‐fabricated cerrobend masks) and treatment speeds of up to < $20\;\mathrm{mm}s^{-1}$.^7^, ^10^, ^27^ With our new system, we extend treatment velocities up to $50\;\mathrm{mm}s^{-1}$ with the possibility of attaining even faster speeds. Since the same image guidance software is used for both the small animal system and the new robotic system shown in this work, and the same CT protocol was followed, image guidance is only then affected by the change in image resolution for locally acquired x‐rays (0.160mm and 0.023mm respectively) and the change in motor accuracy (0.001mm and 0.060mm respectively). The addition of the new dynamic conformal collimator in our system adds much needed utility, however, it also adds a large source of uncertainty (Table 2). Our system, therefore, replicates the existing small animal treatment capabilities on IMBL (image guidance, conformal fields and dynamic treatment delivery) with a minimal impact on treatment accuracy, while greatly extending treatment capabilities with access to 6 DoF alignment, an increased range of motion, faster treatment velocities and larger conformal fields, and importantly, facilitates large animal treatments. Moreover, our proposed margins have the added benefit that the fields are easier for the dynamic collimator to deliver, as larger, smoother fields require less jerk input, resulting in a reduced load on the system.^9^

The results in Figure 5 and Table 1 were impacted by the technical limitations of our analysis. The pixel size used for field comparison (0.25mm) presented as aliasing in the $2\;\mathrm{mm}s^{-1}$ time‐delay data. The time‐delay data was also near the limit of the frame rate of the imaging system (10ms). Despite these limitations, it is obvious that both the systematic and stochastic errors were affected by treatment delivery speed. Since the robotic manipulator is the largest and slowest device in the treatment delivery system, it is most likely the largest contributor to these delivery errors. We present three possible solutions to reducing the errors in the synchronization between the robot and the rest of the treatment delivery system.

(1) In motion systems, a trade off between position and velocity targets is typically made, and systems are designed to achieve the optimal balance between the two for their given application. The current KUKA controller appears to favor meeting positional requirements over constant velocity targets. Such trade‐offs become pronounced near the operating limits of the system, for example at a treatment delivery speed of $2\;\mathrm{mm}s^{-1}$, which is at the minimum limit of the robot controllers current capabilities. This could be potentially fixed by implementing a custom robot controller designed to prioritize meeting velocity targets over positional targets.
(2) In order not to be limited by the robot capabilities, an accurate external tracking system could be used to monitor the robot position in real‐time during treatment. Such a tracking system would permit the robot to become the primary delivery device, allowing all other devices to react to the motion of the robot. A feedback loop on the time scale of 1msto2ms could be achieved for all secondary devices reacting to the positional changes of the robot. This is both potentially cheaper and easier to implement than developing a custom robot controller, although an improved controller would still benefit the treatment delivery greatly. However, we note that this would only serve to retain the planned field shape and ensure it is aligned with the target, it would not correct for any in‐field dose discrepancies introduced by the robot motion. Such discrepancies would need to be separately evaluated as they would become a function of beam height, dose rate, treatment velocity, and would potentially be averaged out during treatment delivery.
(3) KUKA robotics offer a robot sensor interface (RSI) module, allowing near real‐time feedback (4ms cycle time) into the robot controller and greater control over robot trajectories. With appropriate real‐time tracking of the robot, the trajectory could be modified in real‐time to account for velocity variations in the robot motion. This would also enable more complex treatment deliveries by utilizing variable treatment speeds and non‐linear patient trajectories.

It is also important to note that no existing patient positioning system meets the requirements of fast and accurate dynamic motion for synchrotron radiotherapy treatments; thus, a customized solution was required. Therefore, it is difficult to compare our system to existing technologies, as either the use‐case or the implementation of robotic manipulators is far different to our own. For example, industrial robots are very capable of statically positioning objects with great accuracy. Such is the case for the robot in this study, which can achieve static positional accuracies of ± 0.06mm for payloads up to 150kg. The robots used in proton facilities,^11^ and even the CyberKnife system,^13^ are of similar design and capabilities. The dynamic stability of our robot and control system when following a pre‐defined path is also accurate to within ± 0.20mm,^18^ although this may vary between manufacturers, robot designs and control system implementations.

In our work, for an ideal scenario treating a medical physics phantom, we have presented margins of up to 2.3mm for $50\;\mathrm{mm}s^{-1}$ treatment speeds. However, if the systematic timing errors are corrected (using any of the three methods above), our treatment delivery system would be capable of achieving sub‐1.1mm margins, less than the targeting accuracy of the CyberKnife system with a moving target (1.5mm),^12^ patient motion notwithstanding. Herein lies a limitation of our work, in that it does not consider errors contributed by patient motion, contouring or biological considerations. These sources of uncertainty should be investigated as the technique improves and develops.

Aside from immobilization techniques and calibration of the robot for a patient on a bed, in clinical practice patient motion is accounted for by different mechanisms depending on the treatment type. For example, proton therapy can steer the beam to follow the patient motion while the patient positioning robot is held still. Alternatively, the CyberKnife system can dynamically move the source, which is attached to its own robotic manipulator, although this is orders of magnitude slower than the steering of a proton beam. Importantly, the CyberKnife system, which is the only system to dynamically drive a robot during treatment, has a latency of 115ms when reacting to changes in motion^13^ (this is accounted for by utilizing a predictive patient motion model).

While patient motion in the treatment system is beyond the scope of this work, a potential solution is available. Real‐time target tracking could be fed into the proposed KUKA RSI module, and even the dynamic collimator, such that the treatment delivery can be actively modified to compensate for patient motion. This would also require a mechanism to monitor the patient position in real‐time using x‐ray based monitoring^28^ or surface guidance.^29^ Further, additional safety features could be implemented, such as aborting treatment due to patient motion events (such as sneezing or coughing) or even malfunctioning equipment.

## 5 CONCLUSION

We have implemented the first robotic treatment delivery system for synchrotron radiation therapy treatments, incorporating a newly designed dynamic collimator. The treatment system, with all its moving parts, showed both good positional accuracy and repeatability between delivered fields. The largest positional error in field delivery is currently attributed to the timing of the communication between devices, which can be fixed with the proposed future developments. The proposed treatment margins required to utilize this system are acceptable, and so the system is ready to support future treatments on IMBL.

## CONFLICT OF INTEREST STATEMENT

NH receives research grant support from Varian Medical Systems and Reflexion Medical for unrelated work.
